# Combined’ Neck/Back Pain and Psychological Distress/Morbidity Among the Saudi Population: A Cross-Sectional Study

**DOI:** 10.3389/fpsyg.2022.870600

**Published:** 2022-04-19

**Authors:** Sameer Al-Ghamdi, Mamdouh M. Shubair, Khadijah Angawi, Jamaan Al-Zahrani, Abdulrahman Ali M. Khormi, Reem Falah Alshammari, Nawaf Safaq Alshammari, Raed Aldahash, Bander Yahya Otayf, Hayat Saleh Al-Zahrani, Manayir Sultan Aleshaiwi, Khaled K. Aldossari

**Affiliations:** ^1^Family and Community Medicine Department, College of Medicine, Prince Sattam Bin Abdulaziz University, Al-Kharj, Saudi Arabia; ^2^School of Health Sciences, University of Northern British Columbia (UNBC), Prince George, BC, Canada; ^3^Department of Health Services and Hospital Administration, Faculty of Economics and Administration, King Abdulaziz University, Jeddah, Saudi Arabia; ^4^Department of Internal Medicine, College of Medicine, Prince Sattam Bin Abdulaziz University, Al-Kharj, Saudi Arabia; ^5^Family and Community Medicine Department, University of Hail (UOH), Hail, Saudi Arabia; ^6^King Abdulaziz Medical City, Riyadh, Saudi Arabia; ^7^King Abdullah International Medical Research Center (KAIMRC), Department of Medicine, Ministry of National Guard Health Affairs, King Saud Bin Abdulaziz University for Health Science, Riyadh, Saudi Arabia; ^8^College of Medicine, Jazan University, Jazan, Saudi Arabia; ^9^Family Medicine and Medical Education, Princess Nourah Bint Abdulrahman University, Riyadh, Saudi Arabia; ^10^Ministry of Health, Riyadh, Saudi Arabia

**Keywords:** neck pain, back pain, psychological distress, Saudi Arabia, cross-section study

## Abstract

**Background:**

Psychological distress/morbidity is amongst the primary reason for the cause of pain at multiple sites, its progression, and recovery. Though still not very clear if physical pain in the neck or the back may predict psychological morbidities or not. Thus, we investigated the association between combined neck or back pain and psychological distress/morbidity.

**Methods:**

A cross-sectional study was conducted in Al-Kharj, Saudi Arabia, including 1,003 individuals. The questionnaire comprised of General Health Questionnaire-12 (GHQ-12) and some questions about neck and back pain. Data analysis was done using statistical software SPSS version 26.0.

**Results:**

The results of the multivariate analysis revealed a significant positive association between neck/back pain status and total GHQ score (unstandardized Beta = 2.442, *P* ≤ 0.0001). Having neck/back pain had almost a 2.5 times greater risk of psychological distress/morbidity. Further, females were more likely to have a higher risk of psychological distress/morbidity (unstandardized Beta = 1.334, *P* = 0.007) than males while adjusting for sociodemographic and clinical characteristics.

**Conclusion:**

The combination of neck and back pain was significantly associated with the Saudi population’s psychological problems. Therefore, the Saudi government needs to devise high-risk strategies and allocate adequate resources to the cause so that at-risk people can be shielded from the adverse complications arising from this condition in the long run.

## Background

It is anticipated that approximately 65–80% of individuals experience low-back pain (LBP) during their entire life globally (Walker, 2000). The estimated prevalence of neck pain ranges between 20 to 45% in developed countries (Van der Windt et al., 2002; Hoy et al., 2010). The neck pain and backache often result in functional disability and loss of productivity at work (Van der Windt et al., 2002). Further, the existing premise reveals that between 1999 to 2000, pain in the neck and/or back has resulted in around 2.5 million medical appointments each month, thereby leading to higher medical costs in the United States (Stewart et al., 2003). For example, the annual cost of low-back pain is more than $100 billion, mainly due to the lack of yield or productivity and loss of wages (Katz, 2006). More specifically, approximately 149 million days of work annually are missed due to LBP, which results in adverse physical and psychological consequences. For instance, data from developed countries show that neck and shoulder pain substantially impacts people and the population (Van der Windt et al., 2002).

Low-back and neck pain and associated psychological morbidities such as depression are the most common motivations for pursuing medical attention (Hurwitz et al., 2003). It was found that 68.4% of the subjects having neck pain suffer from anxiety while 55.7% of these patients have depression (Elbinoune et al., 2016). Among these subjects, the factors associated with psychiatric morbidities include cervicobrachial neuralgia, low level of education, and severity of the pain (Elbinoune et al., 2016). Despite the established and expected relationship between these common comorbidities, temporal and causal directions and precise mechanisms connecting pain with psychological problems remain indescribable, mainly in developing countries (Rush et al., 2000). Some hypotheses have been put forth to elucidate the association between low back and neck pain with psychological disorders. Such as, one potential hypothesis proposes that low back and neck pain increases the risk of psychological morbidities. Likewise, a second hypothesis could be reversed if psychological morbidity can increase low backache (Hurwitz et al., 2003). These hypotheses have mainly been tested in developed countries, and even though it is supported by whatever little literature is available, there’s little support for the antecedent hypothesis. Also, very few studies have examined its validity in a local setting (Banks and Kerns, 1996; Fishbain et al., 1997). Hence there is a lack of evidence from developing countries on studying the association between combined low back and neck pain and psychological morbidities. While various researchers have explained the association of psychiatric comorbidities with chronic pain, only a few have analyzed the pattern of physical pain sites, such as low back and neck pain, with the psychological status (Hurwitz et al., 2005; Leijon et al., 2009). Consequently, this study aimed at investigating the association of psychological aspects with specific musculoskeletal sites (neck and back, combined) amongst the residents of central region of Saudi Arabia.

## Methods

A cross-sectional study was carried out in Al-Kharj, Saudi Arabia. A total of 1,200 participants from the general population participated in the survey (response rate 86%). This survey timeframe started in January 2016 and was completed in June 2016. A detailed description of methods has been published previously (El-Metwally et al., 2019).

The inclusion criteria were age greater than or equal to 18 years, Saudi nationals, Al-Kharj resident, while non-Saudis, age greater than and equal to 18 years, and any individual with impaired mental health conditions were excluded. Moreover, individuals who did not sign the written consent form were also excluded. All the procedures were explained, before recruitment, to those willing to participate in the study, followed by their verbal and written consent. The data collection was conducted using multistage stratified cluster sampling. A cluster sampling method was used for the data collection from 33 institutes (21 government and 11 private). The investigators took a random number of clusters, whereas, a computer-generated list was applied for the selection of the sample population from these institutions. The sample size was calculated using the following assumptions. The first assumption is that based on previous research the percentage of back pain in the otherwise healthy people in the population is 30%. While the percentage of back pain among patients with psychological morbidities is 55.6% with a confidence level of 95%, a power of 80%.

Proportion 1: 0.3

Proportion 2: 0.556

Confidence level: 0.95

Power: 0.8

Ratio of sample sizes (n2/n1): 0.05

Tails: 2

**Sample sizes required:**

Sample size 1 (n1): 642

Sample size 2 (n2): 33

Total sample size (both groups): 675

After data collection, the incomplete questionnaires were excluded. The final analysis included a total 1,031 of participants. A self-administered questionnaire in the Arabic language (validated) was used for this survey. It comprised multiple sections, including sociodemographic characteristics (age, marital status, education, and employment), questions about chronic neck and back pain, chronic medical conditions, and general health. The last section of the questionnaire consists of a general health questionnaire (GHQ)-12 scale. It was used to inquire about the general mental health of the participants (Montazeri et al., 2003). The scale consists of 12 questions that assessed the severity of psychological distress/morbidity using a four-point Likert scoring technique (0-always to 3-never). The summative score indicates the psychological morbidity of the individual. We had already published the details about the questionnaire in our previous studies (El-Metwally et al., 2019). Any pain that persists consecutively for more than 3 months is categorized as chronic pain (Blyth, 2008). The trained staff takes the height and weight of the participants according to the standard protocols. The height (m) and weight (kg) of the participants were used to calculate their body mass index (BMI) as per the standard formula. Individuals having a BMI < 25 kg/m^2^ were classified as normal, ≥ 25 kg/m^2^ as overweight, while those with a BMI of ≥ 30 kg/m^2^ were classified as obese (CDC, 2020).

Data were tabulated and analyzed with SPSS v.26 (SPSS Inc., Chicago, IL, United States). Pearson’s r (bivariate correlation coefficient) with the total GHQ score of respondents being the continuous variable and the **“combined”** neck and back pain being the dichotomous variable (0 = no and 1 = yes). Moreover, the prevalence of neck/back pain was analyzed as a binary variable as well (0 = no and 1 = yes) and differentiated with sociodemographic factors including gender, job status, and other risk factors (diabetes status; the presence of psychological morbidities such as anxiety/depression; hypertension status; and BMI class). We conducted six (*n* = 6) bivariate cross-tabulation (*X*^2^) tests to evaluate the association between neck/back pain status and each of the variables mentioned earlier. A multiple regression analysis was carried out by regressing the continuous total GHQ score (outcome variable) on the “combined” neck/back pain status (binary variable), sociodemographic, and other variables.

## Results

### Univariate Analysis

The results showed a significant moderate positive association between increased neck/back pain and a higher prevalence of psychological distress (as measured by total GHQ score). The Pearson’s correlation coefficient for this association was *r* = 0.351, *P* < 0.0001.

Our results show that females (64.5%) have a significantly higher prevalence of neck/back pain compared with their male counterparts (35.5%). The Chi-Squared test: (*X*^2^) = 3.102, *P* = 0.045 (Table 1).

**TABLE 1:** Comparison between males and females regarding Neck/Back pain status in the Al-Kharj population sample (*n* = 1,019).

Variables	No neck/back pain (*n* = 957)	Yes neck/back pain (*n* = 62)	Total (*n* = 1019)
	
	*n* (%)	*n* (%)	*n* (%)
**Respondent gender**			
Male	359 (37.5)	22 (35.5)	381 (37.4)
Female	598 (62.5)	40 (64.5)	638 (62.6)
**Total**	957 (100)	62 (100)	1019 (100)

*Chi-Square (X^2^) = 3.102, P = 0.045.*

Regarding job/employment type, a significant proportion of civilian workers have neck/back pain (59.7%) compared with those unemployed (1.6%) or soldiers (38.7%). The Chi-Squared test: (*X*^2^) = 5.238, *P* = 0.03 (Table 2).

**TABLE 2:** Comparison between job type and Neck/Back pain status in the Al-Kharj population sample (*n* = 1,019).

Variables	No neck/back pain (*n* = 957)	Yes neck/back pain (*n* = 62)	Total (*n* = 1019)
	
	*n* (%)	*n* (%)	*n* (%)
**Job type**			
Not working	31 (3.1)	1 (1.6)	32 (3.1)
Civilian	510 (53.3)	37 (38.7)	547 (43.2)
Soldier	416 (43.5)	24 (38.7)	440 (43.2)
**Total**	957 (100)	62 (100)	1019 (100)

*Chi-Square (X^2^) = 5.238, P = 0.03.*

The proportion of diabetic/prediabetic individuals with neck/back pain (38.7%) was significantly higher than that of diabetic/prediabetic individuals who do not have neck/back pain (26.3%). A significant proportion of non-diabetic persons (61.3%) have neck/back pain. The Chi-Squared test: (*X*^2^) = 4.517, *P* = 0.034 (Table 3).

**TABLE 3:** Comparison between diabetic/prediabetic and non-diabetic individuals regarding Neck/Back pain status in the Al-Kharj population sample (*n* = 1,019).

Variables	No neck/back pain (*n* = 957)	Yes neck/back pain (*n* = 62)	Total (*n* = 1019)
	
	*n* (%)	*n* (%)	*n* (%)
**Diabetic status**			
diabetic/prediabetic	252 (26.3)	24 (38.7)	276 (27.1)
non-diabetic	705 (73.7)	38 (61.3)	743 (72.9)
**Total**	957 (100)	62 (100)	1019 (100)

*Chi-Square (X^2^) = 4.517, P = 0.034.*

When neck/back pain status was examined according to the presence or absence of psychological morbidities such as anxiety or depression (Table 4), it was found that 28.4% of individuals who have any anxiety or depression have a significantly higher prevalence of neck/back pain; compared with only 4.7% of their counterparts who do not have neck/back pain. The Chi-Squared test: (*X*^2^) = 64.262, *P* ≤ 0.0001 (Table 4).

**TABLE 4:** Comparison between presence or absence of psychological disease (anxiety; depression) according to Neck/Back pain status in the Al-Kharj population sample (*n* = 1,031).

Variables	No neck/back pain (*n* = 957)	Yes neck/back pain (*n* = 74)	Total (*n* = 1031)
	
	*n* (%)	*n* (%)	*n* (%)
**Psychological disease (anxiety or depression)**			
No	912 (95.3)	53 (71.6)	965 (93.6)
Yes	45 (4.7)	21 (28.4)	66 (6.4)
**Total**	957 (100)	74 (100)	1031 (100)

*Chi-Square (X^2^) = 64.262, P ≤ 0.0001.*

The association between neck/back pain and hypertension status was investigated (Table 5). The results showed that the proportion of hypertensive individuals with neck/back pain (5.4%) is significantly higher than hypertensive individuals who do not have neck/back pain (4.8%). Notably, non-hypertensive individuals with neck/back pain (94.6%) and with no neck/back pain (95.2%) were also significant. The Chi-Squared test: (*X*^2^) = 5.371, *P* = 0.042 (Table 5).

**TABLE 5:** Comparison between hypertension status and Neck/Back status in the Al-Kharj population sample (*n* = 1,031).

Variables	No neck/back pain (*n* = 957)	Yes neck/back pain (*n* = 74)	Total (*n* = 1031)
	
	*n* (%)	*n* (%)	*n* (%)
**Hypertension status**			
Non-hypertensive	911 (95.2)	70 (94.6)	981 (95.2)
Hypertensive	46 (4.8)	4 (5.4)	50 (4.8)
**Total**	957 (100)	74 (100)	1031 (100)

*Chi-Square (X^2^) = 5.371, P = 0.042.*

The relationship of body weight status, assessed by BMI class (categorical variable), and neck/back pain was examined (Table 6). The results revealed no significant difference among the BMI categories regarding the prevalence of neck/back pain. The Chi-Squared test: (*X*^2^) = 4.983, *P* = 0.173 (Table 6).

**TABLE 6:** Comparison between body mass index (BMI) class and Neck/Back status in the Al-Kharj population sample (*n* = 1,018).

Variables	No neck/back pain (*n* = 957)	Yes neck/back pain (*n* = 61)	Total (*n* = 1018)
	
	*n* (%)	*n* (%)	*n* (%)
**BMI class**			
Non-obese (< 25 kg/m^2^)	444 (46.4)	21 (34.4)	465 (45.7)
Overweight (25–29.9 kg/m^2^)	254 (26.5)	18 (29.5)	272 (26.7)
Class I obese (30–34.9 kg/m^2^)	158 (16.5)	11 (18.0)	169 (16.6)
Class II/III obese (≥ 35 kg/m^2^)	101 (10.6)	11 (18.0)	112 (11.0)
**Total**	957 (100)	61 (100)	1018 (100)

*Chi-Square (X^2^) = 4.983, P = 0.173.*

### Multiple Linear Regression Analysis

The results showed a significant positive association between neck/back pain status and total GHQ score (unstandardized Beta = 2.442, *P* ≤ 0.0001). Having neck/back pain had almost a 2.5 times higher risk of psychological morbidity. Females were more likely to have a higher risk of psychological morbidity (unstandardized Beta = 1.334, *P* = 0.007) than males (Table 7).

**TABLE 7:** Multiple linear regression model regressing total GHQ score on neck/back pain status and sociodemographic and other variables (*n* = 1,018).

Total GHQ score	Unstandardized Beta (B)	S.E. of B	Sig.	Standardized B	95% C.I. for Odds ratio	
					Lower	Upper
Neck/back pain status (yes)	2.442	0.677	**0.000**	0.113	1.114	3.771
Diabetes status (diabetic)	–0.611	0.403	0.130	–0.052	–1.403	0.180
Body Mass Index (BMI)	0.019	0.026	0.480	0.024	–0.033	0.071
Age	–0.003	0.024	0.886	–0.006	–0.051	0.044
Gender (female)	1.334	0.490	**0.007**	0.125	0.872	2.295
Education level	–0.184	0.215	0.392	–0.029	–0.607	0.238
Job (not working; or civilian)	–0.310	0.418	0.458	–0.033	–1.131	0.510
Cholesterol level	–0.294	0.201	0.143	–0.049	–0.689	0.100
Smoking status (yes smoker)	0.058	0.298	0.846	0.007	–0.526	0.642

*Bold values indicate the significant P-value.*

## Discussion

The results of this study revealed that patients with combined low back and neck pain could significantly develop psychological distress/morbidities. Pain at multiple sites and psychological anguish is often categorized as primary predictors of wellbeing. Their relationship has been hypothesized to be bi-directional. The transformation of prolonged unresolved mental distress into physical symptoms is quite well known (Bondesson et al., 2018). These results are analogous with several other studies that used GHQ-12 to measure and establish a close association of psychological morbidity with specific pain sites (Benjamin et al., 2000; Pope et al., 2001; Ye et al., 2007; Seibt et al., 2013; Murakami et al., 2017). These findings are comparable with the existing literature, which reveals that back pain increases the likelihood of developing depression and anxiety (Fernandez et al., 2017). The longitudinal study conducted by Fernandez et al. (2017) found a significant relationship between chronic lower back pain and the probability of depression and anxiety symptoms by two times (Fernandez et al., 2017). Moreover, patients with pain at multiple sites such as the lower back and pain in the neck are at higher risk of depressive disorders (Howe et al., 2015). A study conducted in the Saudi population reported that 71% of individuals with pain at multiple sites experience depression (Al-Maharbi et al., 2018). Likewise, a longitudinal study conducted in Netherlands found that combined pain in the low back and neck increased the chances of depression among individuals aged 18–65 years (Gerrits et al., 2012). Another study conducted among primary care patients found that patients with low back pain reported more psychological distress/morbidity with an odds ratio of 1.5 and significant results (Hurwitz et al., 2003). One study found a relationship between psychological distress and spinal pain through a prospective cohort study (Skillgate et al., 2021). Patients with widespread chronic pain had a higher risk of developing mental disorders by three times when compared to the healthy group (Benjamin et al., 2000). Netherlands Mental Health Survey and Incidence Study data demonstrated that those who reported lower back pain had two times higher risk of depression and anxiety at the follow-up of 3 years (van’t Land et al., 2011). A recently conducted systematic review revealed that subjects with neck pain demonstrated elevated levels of depression and anxiety (Liu et al., 2018). Among the young population complaining otherwise medically unexplained symptoms, somatoform disorder varies between 10.7 to 26.8% (Hilderink et al., 2013).

These findings for the association between the combined neck and back pain with psychological depression could be explained by various mechanisms. One of the plausible explanations for such association could be the stimulation of hereditarily determined biochemical elements, neurobiological processes, or common cortical areas could elucidate this multifaceted association by way of shared pathophysiological paths where the pain in different sites can influence depression or anxiety symptoms. Further, lowered descending inhibitory neurotransmitter projections from the brain can impede the gate-control process of pain, with neurotransmitter decrease associated with the pathophysiology of depression or anxiety (Dersh et al., 2002; Goldenberg, 2010). Further, abnormality of the hypothalamic-pituitary-adrenal (HPA) stress axis can produce higher levels of cortisol hormones among depressed or anxious individuals and also among those who report pain at multiple sites in the body, including the back and neck (Vreeburg et al., 2009). Equally, dysregulation of the autonomic nervous system (ANS) due to enhanced sympathetic or reduced parasympathetic activity has also been linked to pain and psychological morbidity (Raison, 2009). Irregularities in both the HPA stress axis and ANS can produce inflammatory markers in pain and psychological morbidity (Camacho, 2013).

In addition to the above findings regarding the association between combined pain in the neck and back with psychological morbidities, we found that females were found to be at higher risk for negative psychological disorders than males. This finding is generally endorsed by other studies in the literature, which have observed that women are more despondent than men (Hybels et al., 2001; Minicuci et al., 2002; Ormel et al., 2002; Blazer, 2003; Samuelsson et al., 2005). One reasonable explanation for women being at higher risk of depression than men could be that females are more persuaded to visit health care facilities and are more likely to be diagnosed with psychological problems than males. In addition, females are also prone to stresses of life due to their reproductive life span and course of life (Albert, 2015; Albert and Newhouse, 2019). Additionally, increased survival among females can make them more vulnerable to developing psychological morbidities than their counterparts. This is because increased life expectancy may increase the exposure of females to risk factors, thereby negative psychological morbidities.

### Strengths and Limitations

This study has several strengths. It is the first study of its type that statistically analyzed the association between psychological distress and neck and back pain. Furthermore, this is the first study to illustrate the possible psychological factors linked with the combined neck and back pain in an Arab cohort. This is particularly important as physical and psychological interaction with distress varies across ethnicities and nationalities (Bierman and Lee, 2018). It is a population-based study that enrolled all institutes in Al-Kharj that use a multistage stratified clustering technique. This arguably makes our sample representative of the entire population of the central region of Saudi Arabia. Moreover, we used GHQ-12 psychometric properties to measure psychological distress (Endsley et al., 2017; El-Metwally et al., 2018). GHQ-12 is extensively used globally as an effective screening method in primary care settings due to its higher sensitivity and specificity (El-Metwally et al., 2018).

Besides these strengths, there is a possibility of undiagnosed clinical comorbid syndromes such as fibromyalgia, osteoporosis, and arthritis since the control for combined neck and back pain was self-reported. This might alter symptomatology and experience of psychological distress. Additionally, GHQ-12 also remains inapplicable in an emergency setting. While it may help predict anxiety-induced chest pain, which often appears with palpitations, elevated blood pressure, and shortness of breath related to the activation of the sympathetic system, it doesn’t rule out the chances of chest pain being of cardiac origin (Webster et al., 2012).

## Conclusion

This study confirms that psychological wellbeing is considerably poorer in patients experiencing combined neck and back pain than those who do not suffer from such a condition. These findings provide valuable insight for screening such patients and exploring the underlying reasons for the prevalence of such conditions in the first place, thus enabling caregivers to provide timely and effective therapeutic to such patients and preventing further deterioration in their psychological wellbeing. Therefore, diagnosis, screening, and treatment need to be regularly integrated into the daily care of such patients. The government of Saudi Arabia also needs to consider the female population at higher risk to prevent them from experiencing long-term negative complications of psychological distress. At the same time, it must also develop high-risk strategies with adequate resources for other vulnerable people groups. There is a need to conduct well-designed longitudinal studies in Saudi Arabia to explore further the causes and impacts of poor mental health on chronic pain and make causal inferences.

## Data Availability Statement

The raw data supporting the conclusions of this article will be made available by the authors, without undue reservation.

## Ethics Statement

The studies involving human participants were reviewed and approved by the Ethical Committee in College of Medicine, Prince Sattam Bin Abdulaziz University. The patients/participants provided their written informed consent to participate in this study.

## Author Contributions

All authors listed have made a substantial, direct, and intellectual contribution to the work, and approved it for publication.

## Conflict of Interest

The authors declare that the research was conducted in the absence of any commercial or financial relationships that could be construed as a potential conflict of interest.

## Publisher’s Note

All claims expressed in this article are solely those of the authors and do not necessarily represent those of their affiliated organizations, or those of the publisher, the editors and the reviewers. Any product that may be evaluated in this article, or claim that may be made by its manufacturer, is not guaranteed or endorsed by the publisher.
